# Effect of school-based educational water, sanitation, and hygiene intervention on student’s knowledge in a resource-limited setting

**DOI:** 10.1186/s12889-021-12279-2

**Published:** 2021-12-11

**Authors:** Ommy Mushota, Aditya Mathur, Ashish Pathak

**Affiliations:** 1 Global Health, Department of Global Public Health, Karolinska Institutet, SE-171 76 Stockholm, Sweden; 2 Department of Pediatrics, R. D. Gardi Medical College, Ujjain, 456006 India; 3 Department of Women and Children’s Health, International Maternal and Child Health Unit, Uppsala University, SE-751 85 Uppsala, Sweden

**Keywords:** Diarrhea, India, water, sanitation, and hygiene (WASH), intervention, adolescents, knowledge

## Abstract

**Background:**

Globally, diarrhea is one of the major causes of under-5 mortality. India accounts for the highest number of childhood deaths from diarrhea globally. Therefore, facilitating the implementation of water, sanitation, and hygiene (WASH)-related interventions in schools and communities is crucial. In the present study, we investigated the effectiveness of a school-based educational WASH intervention in improving students’ knowledge on prevention and management of diarrhea in Ujjain district, India.

**Methods:**

The present pre–post intervention study with a two-stage (schools and classrooms) cluster sampling was conducted on 1,781 students studying in grades: 8^th^–12^th;^ age: 14–19 years) in schools located in Ujjain, Madhya Pradesh, India. The intervention comprised an educational training session using a WASH training module. The means of pre- and post-intervention scores were compared using repeated measure analysis of variance. A multivariate quantile regression model was used to test the correlation between the change in score after intervention and the independent variables. A *P* value of <0.05 was considered statistically significant.

**Results:**

The proportions of students possessing knowledge on the treatment of diarrhea, use of zinc tablets during an episode of diarrhea, and the symptoms and signs of severe pediatric diarrhea were 28%, 27%, and 27%, respectively, before intervention. These proportions increased (*P*<0.001) after the educational intervention to 72%, 73%, and 74%, respectively. The mean post-intervention knowledge score (34.13) was higher than the mean pre-intervention score (15.17) (F = 16513.36, *P*< 0.001). Age was associated with the knowledge score at the 25^th^ and higher quantile (q). Gender exhibited a greater effect at q10^th^. School location was positively associated at q25^th^ and higher. School type was strongly associated at low quantiles (q10^th^ and q25^th^). School medium exhibited a greater association at low quantiles (≤q25^th^).

**Conclusion:**

WASH- and diarrhea-related knowledge among higher secondary school students increased after the educational intervention. Further research is required to evaluate the sociodemographic characteristics associated with change in the knowledge score to better evaluate school-based educational WASH interventions and improve the management and prevention of diarrhea.

**Supplementary Information:**

The online version contains supplementary material available at 10.1186/s12889-021-12279-2.

## Background

Approximately 534,000 children under 5 years (U-5) die every year because of diarrhea [1]. Globally, nearly 1.6 billion children experience childhood diarrhea [2]. The World Health Organization (WHO) has defined diarrhea as loose watery stools occurring thrice or more per day [3]. Diarrhea is usually caused by pathogens that are commonly transmitted through feco-oral pathways [4]. Diarrheal diseases are a common public health problem in most low-and middle-income countries (LMICs) [5]. In 2016, the majority (89%) of U-5 diarrheal deaths occurred in South Asia and sub-Saharan Africa [4, 6]. However, diarrheal deaths have declined substantially (16.6%) from 1.88 million in 2007 to 1.6 million in 2017 [2]. In the past years, efforts have been made to reduce diarrheal diseases through community- and school-based interventions, promotion of exclusive breastfeeding, and rotavirus vaccinations [1]. Despite these efforts, diarrhea continues to be one of the leading causes of global childhood mortality [7].

In India, the number of childhood diarrheal deaths is the highest globally, with over 400,000 cases recorded annually [8]. Diarrheal deaths are more pervasive in rural areas [9]. The efficacy of oral rehydration salts (ORS) and zinc in preventing 69% of diarrhea-associated U-5 mortality has been confirmed [9, 10]. However, only 39% of children with diarrhea in Indian health centers receive ORS and zinc treatments, whereas antibiotics are prescribed to 72% of children presenting with acute diarrhea [10, 11]. Furthermore, nearly 600 million people lack access to safe drinking water, and fewer than 35% of Indian households have access to clean water from their houses [7]. Additionally, 85% of rural Indian household lack access to clean water [7].

School-going children, particularly adolescents, are considered key knowledge carriers, who generally take their learning from school back to their homes and communities [12]. Therefore, investing in adolescents could be an effective strategy to fight against poverty and inequalities. Furthermore, adolescents can be key change drivers in their communities if provided with the right opportunities, information, and tools [13]. Thus, explaining the significance of water, sanitation, and hygiene (WASH) and dissemination of health information to adolescents in schools may effectively improve knowledge on diarrhea management and prevention within communities.

Knowledge, attitudes, and practices (KAP) studies have been conducted in LMICs to understand and prevent diarrhea using school-based interventions [7, 14]. However, only a few intervention studies analyzed the knowledge of higher secondary school students in diarrhea management and prevention by using a WASH educational intervention [15, 16]. Thus, the present study was designed to analyze the effectiveness of WASH interventions in LMICs and provide valuable recommendations to policymakers in the prevention and management of WASH-related morbidities and mortalities in resource-constrained settings. The objective of the study was to understand the effectiveness of a school-based WASH educational intervention on students’ knowledge on prevention and management of diarrhea in Ujjain district, India.

## Methods

### Study design and setting

The present pre–post intervention study with a two-stage (schools and classrooms) cluster sampling was conducted on 1,781 students studying in 8^th^ to 12^th^ grades between July 2018 and December 2018. The study did not use external controls. The study was conducted in Ujjain district, Madhya Pradesh, Central India. Ujjain district has 5 sub-districts, covering 6,091 square km, and is a plateau [17]. The district has a population of approximately 2 million (1,986,864);approximately 61% (616,353) of the population resides in rural areas, with mostly an agrarian economy [17].

### Sample size calculation

To calculate the sample size, a pilot study was conducted on 65 students, in which the students answered 54% of the questions correctly. These 65 students were selected from five schools by convenient sampling. Sample size calculation was performed with one sample comparison of proportion 54%, two-sided alpha of 0.05, and power of 90%. The minimum sample size obtained after calculation was 1,613, to which 10% was added to account for attrition or refusal rate. Thus, the estimated sample size was 1,774. The students who participated in the pilot study were not included in the main study.

### Sampling strategy and data collection

**Fig. 1:**
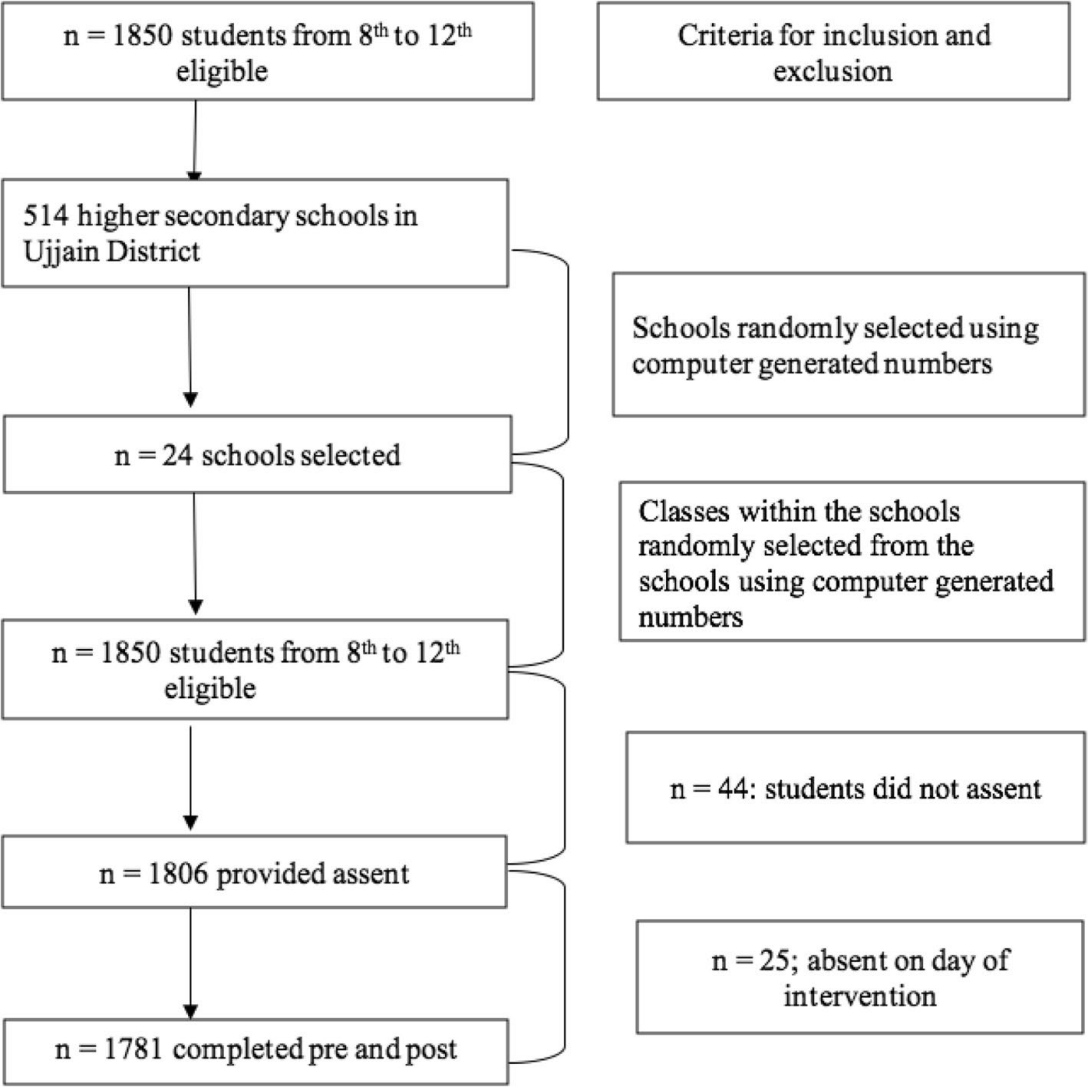
Process of enrolment of participants

A list of public and private higher secondary schools (grades: 8^th^–12^th;^ age: 14–19 years) along with the number of students in each class was obtained from the district education officer. In the first sampling stage, public and private schools were randomly selected from two separate lists of all public and private schools. Figure 1 illustrates the sampling procedure and the inclusion and exclusion criteria for school selection. Of the 514 public and private higher secondary schools in the Ujjain district, 72 schools with at least 40–50 students in each class of 8^th^–12^th^ grade were selected to reduce the number of visits required to obtain the estimated sample size. Of the 72 selected schools, 12 schools from rural areas and 12 schools from urban areas were selected randomly using computer-generated random numbers.

A structured WASH-knowledge questionnaire was developed in English, which was then translated to Hindi according to WHO recommendations for questionnaire translation [18]. The WASH-knowledge questionnaire comprised 15 questions, which were divided into the following four sections: water (1 question), sanitation (5 questions), hygiene (3 questions), and knowledge about diarrhea (6 questions on definition, causes, signs and symptoms, and community treatment) **(Additional file 1: questionnaire in English)**.

The questionnaire also included limited demographic information such as name, age, grade, and gender of the participating students. Each questionnaire required approximately 20 min to complete, with some questions possessing multiple correct options. Each correct answer was given a score of 1. The minimum and maximum possible scores were 0 and 44, respectively. The same questionnaire was used after educational intervention to assess the effect of the intervention. The questionnaires were distributed and collected by 4–6 trained research assistants, who were present in class during the session. Although they assisted the students in understanding the questions in case of any difficulty, they did not assist the students in answering the questions.

### Educational intervention

A visit was scheduled for each school before starting the intervention. Informed written consent was obtained from both the school principal and parents of the students, and written assent was obtained from the students. The trained research assistants ensured that the structured WASH-knowledge questionnaires were completely filled by the students. In case of any missing information, they interviewed the students and filled the missing details. The principal researcher visited the schools to supervise the survey activities. No efforts were made to contact the students who were absent on the day of data collection.

The educational intervention comprised an approximately 60-min practical training session, which included a form of a flip chart and appropriate illustrations and pictures in a Microsoft PowerPoint^TM^ presentation to convey the WASH-related messages to the students. The training module was based on the “Save The Children” community intervention module for childhood diarrhea [19]. Although the training was provided in Hindi in all schools, the medium of instruction in the included schools was either Hindi (regional language) or English. The medium of instruction refers to the language that is used to teach the contents of the educational curriculum.

Two class periods (90-min duration) were required to complete the 20-min pre-intervention questionnaire, whereas the 60-min intervention was provided on the same day. The students were not informed about the post-intervention questionnaire. After a minimum gap of one month following the intervention, the students were asked to complete the same WASH-knowledge questionnaire in 20 min. Overall, 144 sessions were conducted in 6 months, which included 72 pre-intervention and 72 post-intervention sessions.

### Fidelity of intervention

To maintain fidelity in implementation of the intervention, the research assistants received training by the principal investigator. A 4-h training session was conducted to explain the intervention module. All slides in the power point presentation, pictures, and videos were discussed with regards to content and the method of delivering the content. The concepts were reinforced by providing the research assistants an opportunity to engage in role-playing. The training session was repeated once every fortnight during the study period. A training manual was used to articulate the content and delivery of the educational practical session. During the intervention at least two research assistants delivered the intervention, and one senior researcher was present to ensure consistency and fidelity in delivery of the intervention.

### Ethical considerations

The Institutional Ethics Committee of R D Gardi Medical College, Ujjain, India approved the research protocol (IEC-RDGMC-493). Permission was obtained from the District Magistrate, Ujjain to approach the schools and perform the intervention.

### Data management and analysis

Data was collected in schools through paper-based questionnaires which were later entered in Epi Info^TM^ (Version 7.2). Data analysis was done using Stata (Version 16.1, Stata Corp, College Station, Texas, USA). Descriptive statistics were used to determine the proportion of correctly answered questions by the students in the pre- and post-intervention. Pearson χ^2^ was used to test the significance of the difference in proportions at pre- and post-intervention timepoints. For continuous variables, range, mean, and standard deviation was presented. Means of pre- and post-test scores were compared using repeated measures analysis of variance (ANOVA). The effect size of intervention was determined by calculating Cohen’s *d*. Conventionally effect size is considered large if Cohen *d* is greater than or equal to 0.8. Multivariate quantile regression models were used to test the association between difference in pre- and post-intervention score (outcome) and independent variables. The study used quantile regression modeling to capture the full distribution of the outcome-difference in pre- and post-intervention scores, which is superior to an arbitrary binary cut-off for pass or fail. Such a cutoff would be required for binomial regression modeling. The coefficient (b), and 95% confidence interval were estimated for 10^th^, 25^th^, 50^th^ (median), 75^th^ and 90^th^ quantiles of the difference in pre- and post-intervention score based on 500 bootstrap samples. The multivariate quantile regression analysis was performed using the simultaneous quantile regression command in Stata (Version 16.1, Stata Corp, College Station, Texas, USA). A *P* value <0.05 was considered significant.

## Results

A total of 1,806 students completed the pre-intervention questionnaire out of the eligible 1,850. The remaining 44 students did not provide assent. Out of these 1,806 students, 25 students were absent on the day of intervention. Thus, a total of 1,781 participated voluntarily in the study out of the possible 1,806, giving a response rate of 96% (**Figure** **1**). Of the 1,781 participants there were 865 (49%) boys and 916 girls (51%). The mean (±SD) age of the study participants was 15.68 (±1.29) years. **Table** **1** presents the socio-demographic characteristics of the study participants.

**Table 1:** Socio-demographic characteristics of high school students (n=1781) included in the study, Ujjain, India

Variables	n = 1781	%
**Age category**		
14 to 16	1205	68
>16 to 18	466	26
>18	110	6
**Gender**		
Boys	865	49
Girls	916	51
**School location**		
Urban	995	56
Rural	786	44
**School type**		
Private	813	46
Government	968	54
**Predominant language taught in**		
English	678	38
Hindi	1103	62

The mean pre-intervention score was 15.17 and the mean post-intervention score was 34.13 out of maximum possible score of 44. The median pre- and post-intervention scores were 14 and 35, respectively. The effect size of intervention (Cohen’s *d*) was 3.43. The results of repeated measures ANOVA indicated that knowledge score improved post intervention (F = 16513.36, *P*<0.001).

Table 2 shows the proportions of correct answers by the students for definition, causes, signs/symptoms and treatment of diarrhea pre- and post-intervention. It was observed that only about one-third of students had knowledge on the definition of diarrhea and the causes of diarrhea pre-intervention. After the educational WASH intervention, the proportions improved to 72% for both definition and causes, respectively. Improvement post-intervention was also noticed in knowledge of treatment of diarrhea (28% versus 73%; *P*<0.001), use of ORS and Zinc tablets (27% versus 74%; *P*<0.001) and the symptoms/signs of diarrhea (25% versus 75%; *P*<0.001). Further, only 33% of the students had knowledge on benefits of continued breastfeeding in an episode of diarrhea before intervention, this improved to 86% post-intervention (*P*<0.001).

**Table 2:** Number and percentages of correct answers by the students (n=1781) for definition, causes, signs/symptoms and treatment of diarrhea pre- and post-intervention

	Pre intervention	Post intervention	Chi-square	*P* value
	n = 1781	n = 1781		
**Definition of diarrhea**				
Watery stools three or more times a day	559(31)	1394(78)	793.049	<0.001
**What are the causes of diarrhea in a community?**				
Open defecation	555(31)	1392(78)	793.049	<0.001
Not washing hands after defecation	664(37)	1433(80)	678.024	<0.001
Contaminated food/water	574(32)	1371(77)	726.991	<0.001
Germs	609(34)	1353(76)	634.505	<0.001
Flies	548(31)	1333(75)	691.9	<0.001
**What are the symptoms/signs of severe illness in a child with diarrhea?**				
Sunken eyes	567(32)	1392(78)	761.119	<0.001
Slow skin pinch	489(27)	1362(76)	855.766	<0.001
Irritable child	607(34)	1412(79)	733.499	<0.001
Frequent vomiting	581(33)	1404(79)	764.517	<0.001
Difficulty in breastfeeding /eating	547(31)	1285(72)	599.143	<0.001
Dull or becoming unconsciousness	540(30)	1310(74)	690.514	<0.001
Blood in Stool	445(25)	1333(75)	890.25	<0.001
**What treatment should be started on day one of diarrhea?**				
Both ORS and zinc	505(28)	1307(73)	721.175	<0.001
**For how many days should the zinc tablets be taken?**				
Fourteen days	483(27)	1321(74)	786.704	<0.001
**Should a child continue to breastfeed during diarrhea?**				
Yes	579(33)	1535(86)	1037.748	<0.001
**How many steps are there for washing hands?**				
Seven	641(36)	1276(72)	464.478	<0.001

The characteristics of WASH related knowledge of the study participants are presented in Table 3. Lowest proportions of correct answers were observed on questions regarding the use of toilets (25%), washing hands after use of toilet (37%) and benefits of regularly cleaning the toilet (30%) in the pre-intervention, which improved to 77%, 76%, and 72%, respectively post intervention.

**Table 3:** Number and percentages of correct answers by the students (n=1781) for water, sanitation, and hygiene (WASH) related questions pre- and post-intervention

	Pre intervention	Post intervention	Chi-square	*P* value
	n=1781	n=1781		
When is it critical to wash hands with soap and water?				
Before cooking/serving/eating	547(31)	1440(81)	903.257	<0.001
Before feeding/breastfeeding children	610(34)	1346(76)	634.505	<0.001
After defecation	1549(87)	1636(92)	23.693	<0.001
After clearing child’s stool	864(49)	1500(84)	489.533	<0.001
After contact with a sick person	592(33)	1372(77)	696.373	<0.001
After touching animals	472(27)	1293(73)	753.508	<0.001
**Why is a toilet/latrine needed?**				
Use of toilets ensures privacy/security	520(29)	1295(73)	689.685	<0.001
No need to walk very far for defecation	590(33)	1394(78)	729.935	<0.001
It keeps our surroundings clean	680(38)	1359(76)	524.488	<0.001
It helps the old, children and disabled members	615(35)	1357(76)	605.936	<0.001
There is no spread of germs by flies	539(30)	1391(78)	825.739	<0.001
Feces will not be seen in open spaces/sewage	445(25)	1377(77)	963.28	<0.001
**What should be used for washing hands after using the toilet?**				
Soap and water	659(37)	1362(76)	550.939	<0.001
**What are the benefits of regularly cleaning the toilet?**				
The use of toilets increases	542(30)	1282(72)	628.412	<0.001
Flies do not sit on the toilet	700(39)	1372(77)	527.718	<0.001
Surrounding environment remains clean	717(40)	1354(76)	473.631	<0.001
**Where should be the child’s feces be disposed of?**				
Bury in a pit	627(35)	1399(79)	703.192	<0.001
**Which measures can prevent diarrhea?**				
Keeping water pot covered in household	607(34)	1417(80)	768.572	<0.001
Not dipping fingers in glass of drinking water	625(35)	1356(76)	605.936	<0.001
Using utensil with handle to take water from pot.	560(31)	1433(80)	865.468	<0.001
Covering food items	534(30)	1421(80)	899.242	<0.001
Boiling drinking water	592(33)	1278(72)	542.986	<0.001
**Where should we dispose of our household waste?**				
Separating wet and dry garbage in separate boxes	629(35)	1450(81)	773.304	<0.001
In the municipal garbage box/vehicle	532(30)	1396(78)	825.739	<0.001
**Why is it important to keep the house clean?**				
It helps to keep environment clean	799(45)	1480(83)	557.952	<0.001
Flies will not be able to spread germs	691(39)	1369(77)	527.718	<0.001
Children and household members do not get sick often	693(39)	1456(82)	688.805	<0.001

Table 4 reports the results of the multivariate quantile regression analysis of the association between the independent variables and the outcome-difference in pre- and post-intervention scores. Age was associated with knowledge score at 25^th^ and higher quantile; (b) = 0.50; p = 0.004, (b) = 0.60; *P*< 0.001, (b) = 0.65; *P*< 0.001, (b) = 0.87; *P*<0.001. Gender (boys versus girls, with boys as reference) had an impact on the outcome. Boys performed better than girls only at lower quantiles of the outcome (10^th^ and 25^th^). School location (urban versus rural, with urban as reference) also had an impact on the outcome. Urban schools were positively associated with difference in pre- and post-intervention scores at 25^th^ quantile and higher (75^th^ or more), (b) = 1.32; *P* = 0.007, (b) = 2.70; *P*<0.001, (b) = 3.53; *P*<0.001, (b) = 3.98; *P*<0.001. School type (private versus public, with private as reference) was negatively associated with outcome difference in pre- and post-intervention score across all quantiles. School medium (English versus Hindi, with English as reference) was positively associated across all quantiles, a greater association was seen in the lower quantiles (≤ 25^th^ quantile) (b) = 6.70; *P*<0.001, (b) = 6.33; *P*< 0.001.

**Table 4:** Multivariate quantile regression analysis of the association between independent variables and outcome-difference in pre- and post-intervention scores among 1781 high school students in Ujjain, India

	q10		q25		q50 (median)		q75		q90	
	b (95% CI)	***P*** value	b (95% CI)	***P*** value	b (95% CI)	***P*** value	b (95% CI)	***P*** value	b (95% CI)	***P*** value
Age (years)										
Continuous variable	0.34 (-0.21 to 0.89)	0.222	0.50 (0.16 to 0.84)	0.004	0.60 (0.37 to 0.83)	<0.001	0.65 (0.34 to 0.96)	<0.001	0.87 (0.59 to 1.15)	<0.001
Gender										
Boys (ref) Girls	1.94 (0.55 to 3.34)	0.006	1.21 (0.47 to 1.95)	0.001	0.58 (-0.09 to 1.26)	0.090	0.21 (-0.48 to 0.89)	0.554	0.19 (-0.61 to 0.98)	0.648
School location										
Urban (ref) Rural	0.93 ( -0.59 to 2.44)	0.232	1.32 (0.35 to 2.29)	0.007	2.70 (1.74 to 3.66)	<0.001	3.53 (2.67 to 4.40)	<0.001	3.98 (2.93 to 5.03)	<0.001
School type										
Private (ref) Public	-6.95 (-9.61 to -4.29)	<0.001	-6.74 (-7.98 to -5.50)	<0.001	-5.02 (-6.55 to -3.48)	<0.001	-4.73 (-6.03 to -3.44)	<0.001	-4.03 (-5.20 to -2.87)	<0.001
School medium										
English(ref) Hindi	6.70 (3.48 to 9.92)	<0.001	6.33 (4.77 to 7.90)	<0.001	3.85 (2.14 to 5.57)	<0.001	2.97 (1.47 to 4.47)	<0.001	2.26 (0.69 to 3.82)	0.005

q=quantiles, b=beta coefficient, CI=Confidence Interval, ref=Reference, %=percentage

## Discussion

The present study attempted to understand the effectiveness of a school-based WASH educational intervention to teach WASH-based prevention and management of diarrhea to students. The difference in the mean knowledge score between pre- and post-intervention groups was statistically significant (15.17 versus 34.13, respectively; F = 16, 513.36; *P* <0.001; Cohen’s d = 3.43). A Cohen’s d value of >0.8 indicates a large effect size [20]. The intervention resulted in a significant increase in the knowledge score of higher secondary school students on WASH-related factors in the prevention and management of diarrhea.

Prior to the intervention, only a few students were familiar with the treatment of diarrhea, use of zinc tablets during an episode of diarrhea, and the symptoms and signs of severe diarrhea in children. This number increased after the intervention. An educational interventional study conducted in Mumbai, India, related to diarrhea, personal hygiene, and environmental hygiene among school children demonstrated that only 14% of students had awareness about ORS or zinc as treatments for diarrhea. However, the percentage increased to 69% after the educational intervention [21]. The educational intervention in the Mumbai study was implemented in students from the 5^th^ grade aged between 10 and 11 years, and included hygiene-related content. Another WASH intervention conducted in the Philippines in elementary schools used exercises and educational games to teach children about the relationship between personal hygiene and health [14]. The study that a low proportion (21%) of students was aware of ORS or zinc in diarrhea treatment. However, the study reported only a moderate improvement (54%) in the proportion of these students after the intervention [14]. One explanation for the ignorance regarding the use of ORS or zinc as treatment options for diarrhea among the students in the present study may be the lack of knowledge regarding these treatments in resource-poor settings [22]. Studies conducted in resource-poor countries have demonstrated that although communities and healthcare providers in these regions are aware of ORS or zinc, the utilization rates of these treatments are low at both the community and prescriber levels [8, 23, 24]. The knowledge of students on breastfeeding was satisfactory before intervention and improved after intervention. The awareness of the influence of breastfeeding on child health can be attributed to Indian culture, where breastfeeding is universally accepted in both rural and urban areas [25].

The present study exhibited that a high proportion of students was aware of the importance of toilets and latrines in the prevention of diarrhea. This finding is in contrast to that of a study conducted on student's knowledge regarding risk factors for diarrhea in Rwanda, where 11% and 15% of the students reported that drinking dirty water, and poor sanitation and hygiene, respectively, are the causative factors for diarrhea [26]. The findings on the knowledge of hygiene of the students in the present study are consistent with that of a study assessing knowledge, attitude and practice of hygiene among students in Ethiopia [27] . The study reported that 48% of the students exhibited poor knowledge related to hygiene and sanitation. This finding may be due to high illiteracy levels in parents and poor socioeconomic backgrounds [27]. Studies conducted in resource-poor settings have found that communities face financial challenges regarding hardware installation (latrines or toilets), whereas pockets of communities still lack the basic understanding of using a toilet to defecate due to cultural norms [25, 27–32]. Student knowledge regarding the importance of maintaining a clean house and handwashing was comparable to that observed in studies conducted in Kenya and Turkey, which indicates that students are aware of the importance of such practices [15, 33].

Sociodemographic characteristics associated with change in the knowledge score in this study may inform further research. Age changed the outcome in the knowledge score. The score of older students was higher than that of younger students both before and after intervention. This might be because the older students are slightly more knowledgeable than younger students because of experience, education, and cognitive development. This is known as the relative age effect, which is maximum at a younger age. A study in Chile demonstrated that although the relative age effect decreases with the number of years of schooling, it can persist even in 8^th^ grade students [34]. However, the present study did not study the relative age effect. The improvement in the pre- to post-intervention scores in the present study was higher in boys than in girls at lower quantiles (q10^th^ and q25^th^). This finding is in contrast with other studies, which exhibit a clear gender gap in academic achievement, with boys lagging behind [27]. Furthermore, students from urban areas outperformed those from rural areas. A study from Nigeria also reported that the academic performance of students from urban areas was superior to that of students from rural areas [35]. This difference can be attributed to inequalities such as lack of amenities (e.g., roads and hygienic drinking water), sanitation, school infrastructure, teachers, and uninterrupted electricity faced by rural students. The improvement in the pre- to post-intervention scores in the present study was lesser in students from private school compared to those from public schools. This finding was concurrent with that of a literature review on studies conducted in resource-poor countries, which indicated that students from public schools are more likely to work harder and have a more disciplined work ethics than their peers from private schools [28]. The education system in India uses English or other regional languages as the medium of teaching [36]. The improvement in the pre- to post-intervention scores in the present study was higher in students where the medium of instruction was English compared with those schools where medium of instruction was a regional language (Hindi medium). The difference is difficult to explain by traditional thinking which dictates that education becomes more meaningful when learners are able to both receive and act and then transfer and integrate their learning [37]. Therefore, children adapt more quickly to the learning environment if the language of teaching is similar to the one in which they are capable of expressing themselves. Further educational interventions in the regional languages can provide more insights into this aspect.

### Methodological considerations

The strength of the present study is that it focused on assessing the effects of the intervention on adolescents in a resource-constrained setting. Studies have been conducted on KAP of students on hygiene and WASH among schools in LMICs [7, 14]. However, only a few studies have focused on adolescents as knowledge carriers in the prevention and management of diarrhea. Moreover, our study used quantile regression modeling, the advantage of which have been discussed in methods section.

However, our study also has some limitations. The same questionnaire was used during the pre–and post-intervention, which might have allowed students to memorize the questions. The post-intervention assessment was performed 1 month after the intervention. Although no ideal period for the post-intervention assessment is universally accepted, a longer (3- to 6-month) period could have decreased the possibility of question memorization. The present study only included classes with up to 50 students due to the availability of limited resources (time and logistics) for data collection. This could have led to exclusion of some small schools with smaller class sizes, ultimately leading to potential selection bias. The study did not include any controls, which would have made the study design more robust. Follow-up longitudinal studies are warranted to evaluate the long-term effects of educational interventions on diarrhea and WASH-related outcomes and to assess whether the improved knowledge among adolescents translates into meaningful behavioral changes in their communities.

## Conclusions

A significant increase in WASH- and diarrhea-related knowledge can be achieved among higher secondary school adolescents after an educational intervention. The effect size of the intervention was large. As diarrhea continues to be one of the leading causes of U-5 mortality, people-centered interventions are required to effectively manage and prevent diarrhea in resource-poor settings. The study has crucial policy implications such as use of adolescents as knowledge carriers for WASH-related community interventions. Future research should focus on long-term follow-up to better evaluate knowledge retention and assess whether the enhanced knowledge translates into meaningful behavioral change in communities.

## Supplementary Information

**ESM 1.**

## Data Availability

The dataset used and/or analysed during the current study is available from the corresponding author on reasonable request. Individual data can due to confidentiality reasons not be made public. All enquiries regarding data sharing should be made to- The Chairman, Institutional Ethics Committee, R D Gardi Medical College, Agar Road, Ujjain, India 456006 (E-mail uctharc@sancharnet.in). The name of data set corresponding to the study is School_dia_intervention data.

## List of Abbreviations

**ANOVA**: Analysis of Variance
**DEO**: District Education Officer
**GBD**: Global Burden of Disease
**KAP**: Knowledge Attitudes and Practices
**LMICS**: Low- and Middle-Income Countries
**ORS**: Oral Rehydration Salts
**SSA**: Sub Saharan Africa
**UNICEF**: United Nations Children’s Emergency Fund
**U-5**: Under Five
**WASH**: Water Sanitation and Hygiene
**WHO**: World Health Organization
