# Supplementary material for: Effect of school-based educational water, sanitation, and hygiene intervention on student’s knowledge in a resource-limited setting
Source: BMC Public Health. 2021 Dec 11;21:2258. doi: 10.1186/s12889-021-12279-2 (PMC8666030; doi:10.1186/s12889-021-12279-2)
Supplement: Supplementary file 1 — ESM 1. [file 12889_2021_12279_MOESM1_ESM.docx]

# Questionnaire English

**DIARRHEA RELATED QUESTIONNAIRE (PRE-POST TEST) FOR STUDENTS**

**School Name ______ Student name _______ Sex ____ Class ______ Section ___**

**Total marks 44**

**Correct answers are highlighted in green**

1. What is the definition of diarrhea? Maximum marks – 1 *Definition, cause, sign/symptom & treatment*

| 1. Watery stool one time in a day, | 2. Watery stool two times in a day, |
| --- | --- |
| 3. Watery stools three or more times a day, | 4. Watery stool more than five times |
| 5. Don’t know) |  |

2. What are the causes of diarrhea in a community? **(You can mark more than one answer)** Maximum marks – 5 *Definition, cause, sign/symptom & treatment*

| 1. Open defecation | 2. Not washing hands after defecation |
| --- | --- |
| 3. Contaminated food/water | 4. Eating spicy food |
| 5. Hot weather | 6. Germs |
| 7. Flies | 8. Don’t know |

3. What are symptoms or signs of severe illness after diarrhea? **(You can mark more than one answer)** Maximum marks – 7 *Definition, cause, sign/symptom & treatment*

| 1. Sunken eyes | 2. Slow skin pinch |
| --- | --- |
| 3. Irritable child | 4. Frequent vomiting |
| 5. Difficulty in breastfeeding / feeding /eating | 6. Dull or becoming unconsciousness |
| 7. Blood in Stool | 8. Don't know |

4. When is it important / critical to wash hands with soap and water? **(You can mark more than one answer)** Maximum marks – 6 *Hygiene*

| 1. Before cooking/serving/eating | 2. Before feeding/breastfeeding children |
| --- | --- |
| 3. After defecation | 4. After clearing child’s stool |
| 5. After coming in contact with a sick person | 6. After touching animals |
| 7. Don’t know |  |

5. Why according to you toilet/latrine is needed? **(You can mark more than one answer)** Maximum marks – 6 *Sanitation*

| 1. Use of toilets ensures privacy/security | 2.No need to walk very far for defecation |
| --- | --- |
| 3. It keeps our surroundings clean | 4.It helps the old, children and disabled members |
| 5. There is no spread of germs by flies | 6.Feaces will not be seen in open spaces/sewage |
| 7. Don’t know | 8.Other |

6. What should be used for washing hands after using toilet? Maximum marks – 1 *Hygiene*

| 1. Only water | 2.Ash |
| --- | --- |
| 3. Mud | 4.Soap and water |
| 5. Don’t know | 6.Other |

1

7. What are the benefits of regularly cleaning the toilet? **(You can mark more than one answer)** Maximum marks – 3 *Sanitation*

| 1. Use of toilets increases | 2.Flies do not sit on the toilet which prevents disease spread |
| --- | --- |
| 3. Surrounding environment remains clean | 4.Don't know |
| 5. Other |  |

8. Where the child’s feces should be disposed off, if there is no toilet? Maximum marks – 1 *Sanitation*

| 1. Bury in a pit | 2. Throw in open area / space |
| --- | --- |
| 3. Drain it in open sewage | 4. Don’t know |
| 5. Other |  |

9. Diarrhea can be prevented by which of the following measures? **(You can mark more than one answer)** Maximum marks – 5 *Water*

| 1. Keeping water pot covered in household | 2. Not dipping fingers in glass of drinking water |
| --- | --- |
| 3. Using utensil with handle to take water from pot. | 4.Covering food items |
| 5. Boiling drinking water | 6.Other |
| 7. Don’t know |  |

10. Where should we dispose our household waste? **(You can mark more than one answer)** Maximum marks – 2 *Sanitation*

| 1. Separating wet and dry garbage in separate boxes | 2.Anywhere outside home in open |
| --- | --- |
| 3. Specified place outside home | 4. Burning the waste near home |
| 5. Garbage should be buried in a pit | 6. In the municipal garbage box/vehicle |
| 7. Don’t know | 8.Other |

11. Why is it important to keep cleanliness around the house? **(You can mark more than one answer)** Maximum marks – 3 *Sanitation*

| 1. It helps to keep environment clean | 2. Flies will not be able to spread germs |
| --- | --- |
| 3. Children and household members do not get sick often | 4.Don't know |
| 5. Other |  |

12. What treatment should be started on day one of diarrhea? Maximum marks – 1 *Definition, cause, sign/symptom & treatment*

| 1. ORS | 2. Zinc |
| --- | --- |
| 2. Both ORS and zinc | 4. Don’t know |

13. For how many days zinc tablets should be taken? Maximum marks - 1 *Definition, cause, sign/symptom & treatment*

| 1. One to two days | 2. Three days |
| --- | --- |
| 3. Five days | 4. Fourteen days |
| 5. Don’t know |  |

14.Should a child continued to breastfeed during diarrhea? Maximum marks – 1 *Definition, cause, sign/symptom & treatment*

| 1. Yes | 2. No |
| --- | --- |
| 3. Don’t know |  |

15. How many steps are there for washing hands? Maximum marks - 1 *Definition, cause, sign/symptom & treatment*

| 1.7 | 2. 8 |
| --- | --- |
| 3. 9 | 4.10 |
| 5. Don’t know |  |
